# Electrocochleography in the diagnosis of third window conditions

**DOI:** 10.3389/fneur.2023.1263513

**Published:** 2024-01-04

**Authors:** Paul R. Kileny, Megan M. Cherry, Devin L. McCaslin

**Affiliations:** Michigan Medicine, Department of Otolaryngology, Head and Neck Surgery, Division of Audiology, University of Michigan, Ann Arbor, MI, United States

**Keywords:** electrocochleography, SSCD = superior semicircular canal dehiscence, air-bone gap, SP/AP ratio, autophony, bone conduction hypersensitivity, diagnosis, EcochG

## Abstract

**Introduction:**

Superior semicircular canal dehiscence (SSCD) is the best-known and most common presentation of so-called “third window conditions.” There are a variety of diagnostic measures and tests for this condition in the current literature, including air-bone gap, vestibular-evoked myogenic potentials, and electrocochleography (EcochG). The purpose of this study was to investigate the diagnostic utility of EcochG and its relationship to air-bone gap in a cohort of patients with confirmed SSCD.

**Methods:**

We reviewed data from 20 patients (11 female and 9 male subjects, age ranging 21–78 years), with confirmed unilateral or bilateral superior canal dehiscence. In total, 11 patients had unilateral SSCD and 9 patients had bilateral SSCD as determined by high-resolution CT scan. This resulted in the inclusion of twenty-nine ears with superior canal dehiscence and 11 normal ears.

**Results:**

Our results indicated that all confirmed SSCD ears presented with an abnormal EcochG SP/AP value and that there was a statistically significant difference between normal and dehiscent ears. There was no statistically significant relationship between air-bone gap and SP/AP ratio in the ears diagnosed with SSCD nor was there a significant difference between dehiscent and normal ears in terms of air-bone gap at three frequencies.

**Discussion:**

These results are consistent with previous studies showing the diagnostic utility of EcochG for this condition and the variability of air-bone gap. While an unexpected air-bone gap continues to be a red flag for SSCD, its absence along with the presence of subjective symptoms is a reasonable indicator for further clinical investigation to include EcochG.

## Introduction

Superior semicircular canal dehiscence (SSCD) is the best-known and most common presentation of so-called “third window conditions” and was first described by Minor et al. (1). A normal inner ear has two openings or “windows” in the otic capsule (i.e., round and oval windows). This condition is characterized by a combination of auditory and vestibular symptoms resulting from this super numerary opening into the otic capsule caused by the absence of bone overlying the superior semicircular canal and, at times, less frequently, other canals such as the posterior canal. Among auditory phenomena, there can be autophony, pulsatile tinnitus, aural fullness, and air-bone gaps in the absence of middle ear pathology. This air-bone gap is typically caused by what is referred to as “bone conduction hyperacusis,” that is, sub-zero dB bone conduction thresholds. When bone-conducted stimuli are used, the third window changes the acoustic impedance on the vestibular side of the cochlear partition resulting in increased auditory sensitivity or lower bone conduction thresholds than would be expected (2). The low-to-mid-frequency air-bone gap is considered to be caused by the dissipation of acoustic energy through the abnormal, supernumerary third window which falsely elevates the air conduction thresholds, and this is added to the bone conduction hyperacusis characterized by unusually low bone conduction thresholds.

Vestibular symptoms may include sound and pressure-induced vertigo, disequilibrium, and, at times, oscillopsia.

The clinical utility of vestibular-evoked myogenic potentials (VEMPs) in identifying and documenting whether dehiscence is “active” has been well documented. The two most commonly described VEMPs are the cervical VEMP (i.e., cVEMP) and the ocular VEMP (i.e., oVEMP). The primary generator of the cVEMP is the saccule, and it is most commonly recorded from the sternocleidomastoid muscle. The oVEMP response is generated by the utricular macula and recorded, most robustly, from the contralateral extraocular muscles (i.e., inferior oblique). Overall, it has been reported that cVEMPs and oVEMPs have a high sensitivity and specificity for identifying SSCD. Hunter et al. reported that when using a criterion of a cVEMP being present at <70-dB nHL threshold, the sensitivity was 73% and the specificity was 94%. The sensitivity was 78.6% for oVEMP amplitudes, exceeding 12 μV, and the specificity was 81.7% (3).

The diagnostic test largely considered the gold standard for identifying SSCD is high-resolution CT. Although it is convenient to think of the bony defect as always occurring at the arcuate eminence, there are data showing that there is substantial variability in where the dehiscence is located (4). The bony defect can occur on the medial descending limb, lateral ascending limb, and the descending limb close to the superior petrosal sinus. Although CT is considered the most sensitive and specific approach to identifying dehiscence, it is not perfect. This is due to the fact that anatomical variations such as a thinning of the bone over the canal can be read as a positive result.

In addition to air and bone conduction audiological testing, computed tomography (CT) of the temporal bone, vestibular-evoked myogenic potentials (VEMP), and electrocochleography (EcochG) have been determined to be diagnostically and interventionally useful and informative for SSCD. Our group was the first one to recognize the utility of EcochG and its contribution to the diagnostic study of superior semicircular canal dehiscence (5).

EcochG is defined as the recording of cochlear potentials such as the cochlear-microphonic (CM) and the summating potential (SP) and the whole nerve action potential (AP) generated by the cochlear nerve. These components of the auditory evoked response are obtained by applying recording techniques that emphasize these particular components. Briefly, EcochG is the measurement of the above defined combination of inner ear and cochlear nerve-generated potentials elicited by a transient acoustic stimulus, i.e., in our case, a click. Responses are recorded using the hydrogel-tipped recording electrode in contact with the lateral surface of the tympanic membrane, introduced with microscopic visualization of the ear canal and the tympanic membrane. Depending upon the polarity configuration of the transient acoustic stimulus, one can record the cochlear microphonic if using constant polarity or the summating potential if using alternating polarity transient stimuli such as clicks. This is followed by the cochlear nerve action potential also referred to as N1 or AP. The summating potential is essentially a direct current potential that follows the stimulus envelope and would be superimposed upon the cochlear microphonic baseline shift when using tone bursts as stimuli. With a click, it is a relatively brief peak or shoulder-like manifestation preceding the cochlear nerve action potential (AP). The summating potential rises from direct current intracellular potentials and is generated predominantly by inner hair cells with some contribution from the outer hairs. The SP is related to a rectified and smooth version of the basilar membrane’s displacement (6). When using a transient stimulus like a click, elicited by alternating polarity stimulation, the summating potential is a short-duration peak preceding the action potential by up to 1 millisecond, and at times, it can blend into the leading edge of the action potential. The action potential (AP) is the cochlear nerve compound action potential and is in fact the manifestation of wave I of the auditory brain stem response. There has been extensive literature regarding the utility of EcochG in the diagnosis of Meniere’s disease for diagnostic purposes and in monitoring the effects of treatment. Based on a previous study by Davis et al. in the 1950s, the position of the basilar membrane that would be displaced when endolymphatic hydrops are present does have an effect on the amplitude of the SP by enhancing it. When the basilar membrane is statically displaced toward the scala of tympani, this results in an increase of the SP/AP ratio due to an increase in the absolute amplitude of the SP, and this has been used in assisting with the diagnosis of Meniere’s disease (7).

In 2009, our team was the first one to identify the presence of an abnormal, elevated SP/AP ratio in patients with confirmed SSCD (5). In this initial report, we presented seven adult patients with unilateral SSCD and four adult patients with bilateral SSCD. All of these patients underwent tympanic electrocochleography. They also underwent air and bone conduction audiology testing, vestibular-evoked myogenic potential testing (VEMP) as well as high-resolution temporal bone computer tomography which was reformatted to optimize the resolution of the superior semicircular canal. At the time, 5 patients of the total 11 patients underwent superior canal occlusion. We also obtained postoperative EcochG from four patients at the time of publication. Additionally, EcochG was monitored continuously during the surgical repair. Of a total of 15 ears with confirmed SSCD using high-resolution CT, 14 were found to have an elevated SP/AP ratio exceeding a value of 0.4, which was the clinical normative limit that has been used in our clinic for the past 3 decades. The mean SP/AP ratio for the affected ears was 0.73, with a standard deviation of 0.29, and for the unaffected ears, it was 0.27, with a standard deviation of 0.14. In terms of audiometry, the air-bone gap was typically the largest at the lowest frequencies in the affected ears, and the mean air conduction threshold was elevated across the frequency range in the affected ears. In the next publication by our group, we reported on 33 patients (45 ears) with clinical symptoms and CT evidence of SSCD. All patients underwent electrocochleography and 8 patients underwent intraoperative electrocochleography during superior canal occlusion as well. Nine patients also underwent postoperative EcochG after occlusion surgery. Overall, electrocochleography was associated with 89% sensitivity and 70% specificity relative to SSCD based on the computer tomography gold standard. The mean SP/AP ratio among affected ears was significantly higher than among unaffected ears: 0.62 vs. 0.29 mean values. This difference was statistically significant. During surgery, the SP/AP ratio increased upon the exposure of the canal lumen, and after completion of occlusion, the SP/AP ratio was reduced to values below the intraoperative baseline with a mean change of 0.23 (standard deviation of plus or minus 0.5). In all patients who underwent postoperative electrocochleography between 1 and 3 months following SSCD repair, the SP/AP ratio was well within normal limits, under the value of 0.4. This was represented by the 9 patients who underwent postoperative outpatient EcochG at the time of that publication (8).

These two early reports convinced us of the diagnostic utility of EcochG in patients suspected of SSCD. Additionally, these early clinical studies also demonstrated the intraoperative monitoring utility of electrocochleography during SSCD repair. Therefore, we adopted EcochG as a clinical diagnostic standard in patients suspected of SSCD and in intraoperative monitoring standards in patients undergoing SSCD repair. More recently, our team demonstrated the strong correlation between EcochG SP/AP ratio normalization and symptom improvement in patients undergoing SSCD repair (9). This study included 46 patients who underwent SSCD repair (14 were unilateral and 6 were bilateral), with 24 who underwent the middle cranial fossa approach and 28 who underwent the transmastoid approach. There were no differences between the surgical groups in terms of preoperative, intraoperative, or postoperative EcochG SP/AP ratio. All patients, regardless of surgical approach, demonstrated subjective improvement in the vestibular symptoms in both groups. Vestibular symptoms persisted more often in patients with concomitant migraine and in a few patients with persistently abnormal EcochG measures postoperatively. Overall, the normalization of the preoperative EcochG values postoperatively correlated with patients experiencing symptom improvement after SSCD repair.

The purpose of the present study was to further explore whether the size of the air-bone gap is associated with EcochG SP/AP ratio values in ears with confirmed SSCD.

## Methods

EcochG was performed by positioning a hydrogel-tipped electrode onto the lateral surface of the tympanic membrane. This was accomplished by direct visualization of the ear canal and tympanic membrane with an oto-microscope. The tympanic membrane electrode was stabilized by the foam tip of the insert audio transducer. Alternating polarity clicks of 100 ms duration presented at 85 to 95 dB nHL were used as stimuli. This range was used subject to magnitude of hearing loss in an effort to obtain clear, repeatable, and interpretable records. Typically, two replications of averaged responses elicited by 1,000 to 2,000 clicks at a rate of 9 to 11.7 per second were obtained and grand averaged. Responses were band-passed filtered (20–1,500 Hz) and averaged, and the SP/AP ratio was calculated (10, 11). As previously mentioned, an SP/AP ratio of greater than 0.4 was considered to be abnormal for the purpose of this study, based on our clinical norms that we have used for approximately 3 decades. All subjects underwent audiological testing consisting of air and bone conduction audiometry, word recognition scores, and speech reception threshold. The speech audiometry results were irrelevant for this patient population, and for the purposes of this study, therefore those results were not included in the data analysis. Every effort was made to determine the presence of sub-zero dBHL bone conduction thresholds. The air-bone gap was evaluated at 250, 500, and 1,000 Hz for each patient to capture this predominantly low-to-mid-frequency phenomenon.

### Subjects

For the purpose of this study, we reviewed data from 20 patients (11 female and 9 male patients) with confirmed unilateral or bilateral superior canal dehiscence. The age range was 21 to 78 years.

Twenty-nine ears with superior canal dehiscence and 11 normal ears were included in the study. In total, 11 participants had unilateral SSCD and 9 patients had bilateral SSCD as determined by high-resolution CT scan. The degree of hearing loss for ears with SSCD ranged from normal hearing thresholds to moderate hearing loss.

Table 1 is a summary of subjects and affected ears. Table 2 lists the participants with age range, gender, air-bone gap values at three frequencies, and EcochG SP/AP values of the affected ears. Table 3 provides a description of normal contralateral ears from our sample.

**Table 1:** Subject demographic information.

	*n*	%
Total participants	20	
Female	11	55%
Male	9	45%
Unilateral	11	55%
Bilateral	9	45%
Total ears	29	
Right ears	13	45%
Left ears	16	55%
Unilateral	11	38%
Bilateral	18	62%

**Table 2:** Individual clinical data used in data analysis.

Participant number	Gender	Age range	ABG 250	ABG 500	ABG 1000	SP/AP	Ear
1	M	40–49	15	5	5	0.5	R^N^
			10	5	5	0.57	L^N^
2	F	40-49	0	0	5	0.61	R
			35	20	25	0.83	L^T^
3	M	50–59	25	15	20	0.6	R
			25	20	15	0.8	L^AT^
4	F	50–59	5	5	15	0.61	R
			5	5	15	0.68	L^A^
5	M	60-69	40	35	35	0.53	R
			20	0	15	0.53	L^AT^
6	M	40–49	10	0	5	0.646	R^N^
			10	0	10	0.525	L^N^
7	F	40-49	5	10	0	0.51	R^A^
			0	10	0	0.44	L
8	F	50–59	35	15	20	0.51	R^T^
			15	10	10	0.55	L
9	F	60–69	45	20	20	0.45	R^T^
			15	0	10	0.5	L^A^
10	F	70–79	5	10	5	0.47	L
11	F	40–49	10	15	5	0.54	L
12	F	30–39	10	10	10	0.66	L
13	M	60–69	10	5	5	0.61	R
14	M	20–29	5	5	10	0.65	L
15	F	40–49	10	5	20	0.41	R
16	M	20–29	10	0	5	0.53	R
17	M	50–59	15	5	15	0.5	L
18	F	30–39	15	5	15	0.596	L
19	F	40–49	5	0	5	0.62	L
20	M	60–69	0	10	10	0.55	R

ABG = Air-Bone Gap; SP/AP – SP/AP Ratio; R = Right; L = Left. ^A^ = Stronger side for autophony. ^T^ = Stronger side for tinnitus/muffled hearing. ^N^ = No lateralizing symptoms.

**Table 3:** Data from contralateral ears (normal).

Participant number	Gender	Age range	ABG 250	ABG 500	ABG 1000	SP/AP	Ear
2	F	70–79	15	20	0	0.35	R
3	F	40–49	0	10	15	0.42	R
6	F	30–39	15	5	5	0.53	R
7	M	60–69	10	5	5	0.27	L
8	M	20–29	5	5	10	0.33	R
11	F	40–49	15	10	15	0.22	L
12	M	20–29	5	0	5	0.24	L
13	M	50–59	10	5	10	0.5	R
16	F	40–49	15	0	10	0.22	R
17	F	40–49	5	0	5	0.31	R
19	M	60–69	−5	10	0	0.35	L

ABG, Air-Bone Gap; SP/AP – SP/AP Ratio; R = Right L = Left.

### Statistical analysis

Continuous variables were presented with means, standard deviations (SD), and ranges; categorical variables were summarized with percentages and frequency counts. Comparisons of variables between ears with and without SSCD were evaluated using Mann–Whitney U tests and linear mixed effects model analysis. All tests were two-sided, and *p*-values of <0.05 were considered statistically significant. Associations of SP/AP ratios with ABGs at 250, 500, and 1,000 Hz were evaluated using Pearson product–moment correlation coefficients. Results were considered significant at the *p* < 0.05 level. Statistical analyses were performed using IBM SPSS Statistics version 29.

## Results

Associations of conductive hearing loss at three frequencies and their average as well as measurements of SP/AP ratios derived from the EcochG among all 29 abnormal ears and 11 normal ears are summarized in Tables 2, 3. In six of the nine patients with bilateral SSCD, the symptoms lateralized to one side. In two of these six patients, tinnitus was the only lateralizing symptom. In an additional two of these six patients, autophony was the only lateralizing symptom. Two patients noted both tinnitus and autophony lateralizing to the same side. Additionally, there was one patient who noted tinnitus lateralized to one side (right) and autophony lateralized to the other side (left). Of the six patients who had lateralizing symptoms, the side with the stronger symptoms had the higher SP/AP ratio for four of the six patients. One patient had equally elevated SP/AP ratios bilaterally. Interestingly, the patient with stronger autophony in the right and stronger tinnitus in the left had a slightly higher SP/AP ratio in the left ear although these values (0.47 and 0.50, respectively) were not significantly different. For the two patients without lateralizing symptoms, the SP/AP ratios differed by 0.07 for one patient and 0.121 for the other patient.

**Figure 1:**
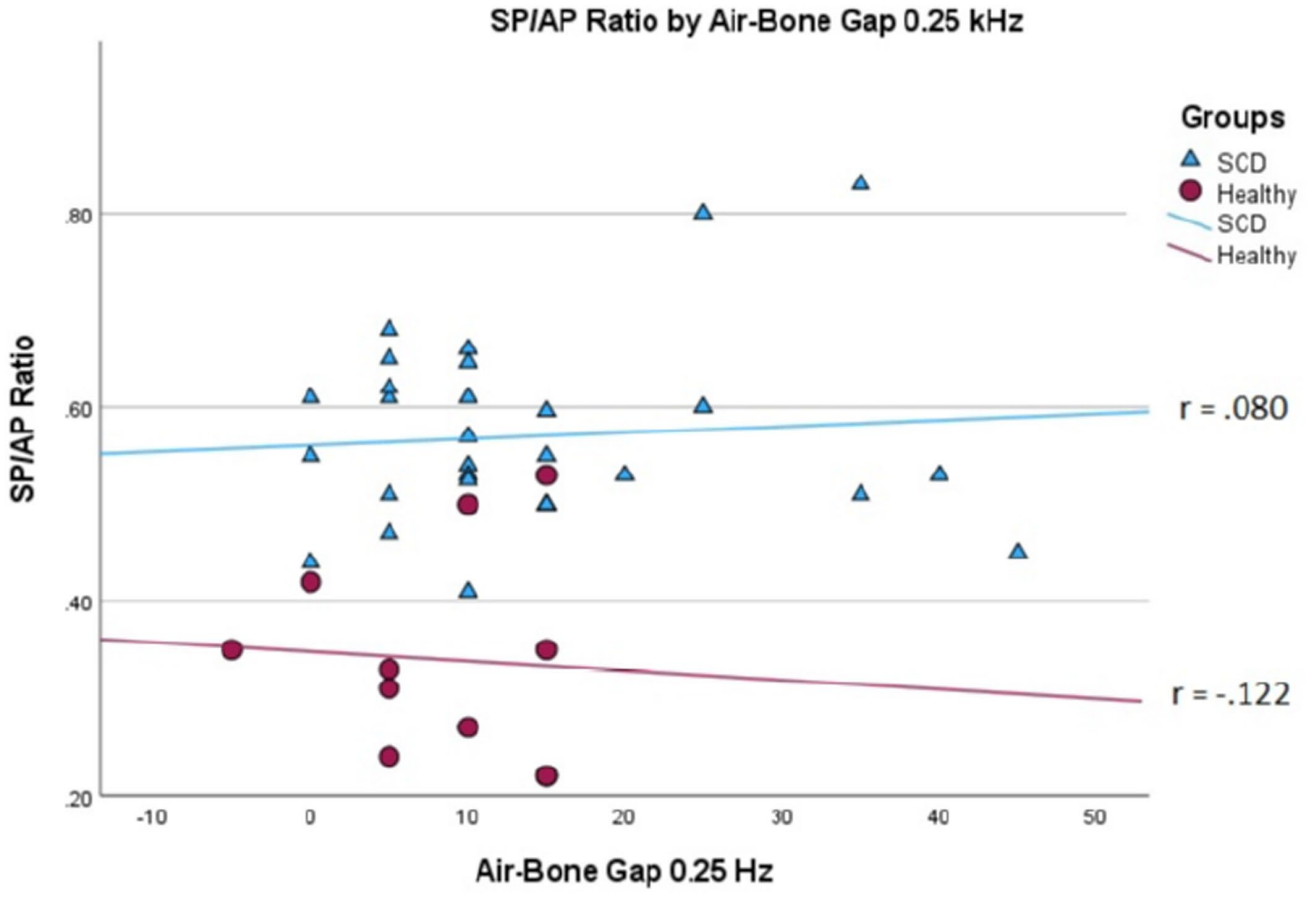
Correlations of ABG at 250 Hz and SP/AP ratio in healthy (Pearson *r* = −0.122, *p* = 0.720) and SSCD (Pearson *r* = −0.080, *p* = 0.678) ears.

**Figure 2:**
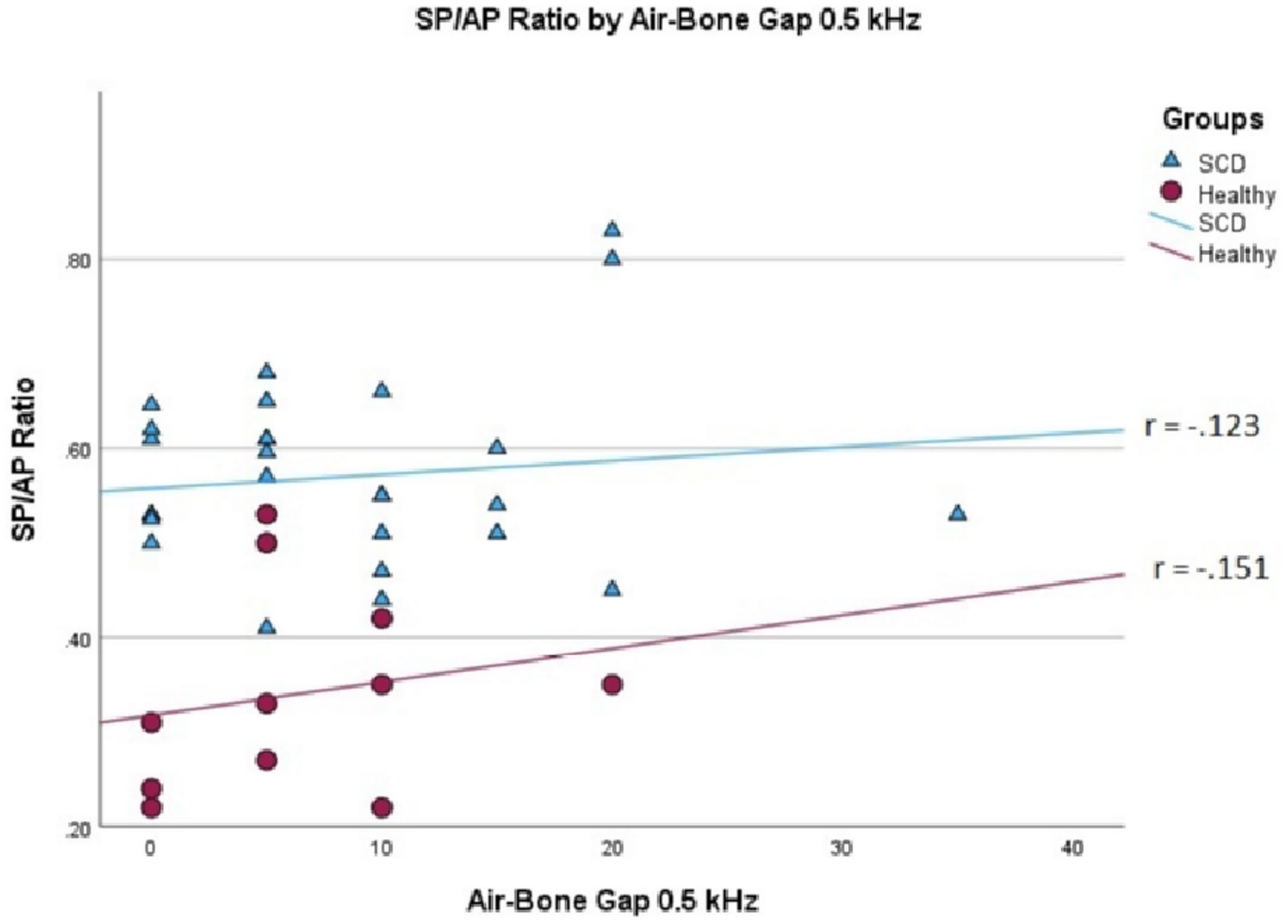
Correlations of ABG at 500 Hz and SP/AP ratio in healthy (Pearson *r* = −0.151, *p* = 0.658) and SSCD (Pearson r = −0.123, *p* = 0.524) ears.

**Figure 3:**
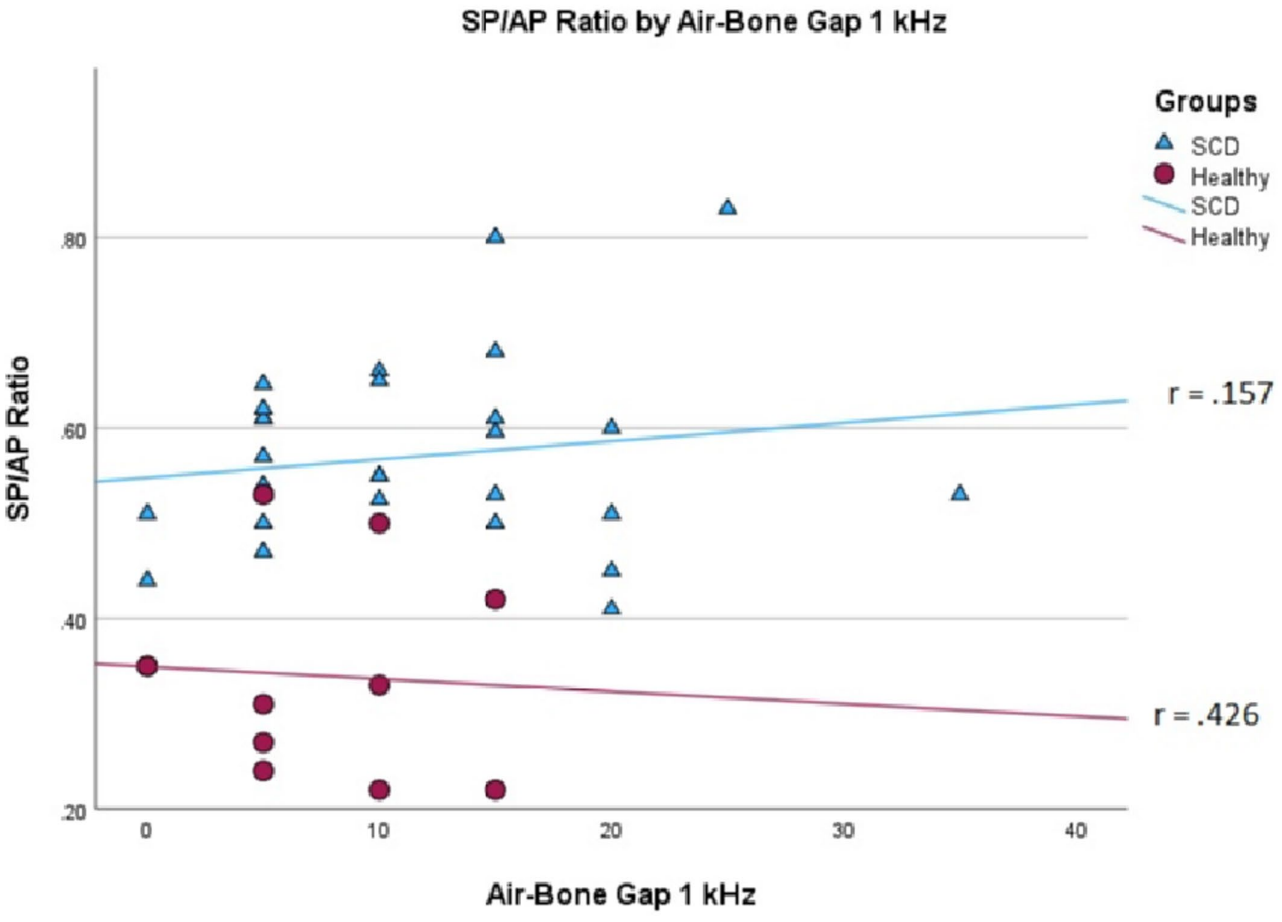
Correlations of ABG at 1000 Hz and SP/AP ratio in healthy (Pearson *r* = −0.426, *p* = 0.192) and SSCD (Pearson *r* = −0.157, *p* = 0.415) ears.

We conducted a linear mixed effects analysis with SP/AP amplitude ratio as the dependent variable. The fixed effect of interest was ears with SSCD and healthy ears and the random effect included subjects. There was a significant main effect of SSCD on SP/AP amplitude ratio (*p* < 0.001). The 29 ears with SSCD (*M* = 0.57, SE = 0.15) had larger SP/AP ratios than the ears with no SSCD from patients with unilateral SSCD (*M* = 0.34, SE = 0.23) indicating a significant increase in the SP/AP ratio in ears with radiologically confirmed SSCD. There was no observed significant correlation between SP/AP amplitude ratio and air-bone gap at any of the tested frequencies. There was no significant difference between SSCD and healthy ears for air-bone gap at any of the frequencies tested. Finally, there was no evidence to suggest that there was a significant positive or negative association of SP/AP amplitude ratio with ABG (Figures 1–3).

### Case study

The purpose of this case study is to illustrate our standard protocol for patients with SSCD, including our EcochG technique, and to provide an example of EcochG waveforms obtained. This is the case of a male (Case 14, in Table 2) with left ear symptoms that were first noted approximately 10 months prior to our consultation. These consisted of hearing his eyes move in his head in his left ear in quiet as well as left-sided pulsatile tinnitus when his head was on the pillow left eat down, left ear autophony, and sound sensitivity. The patient denied diminished hearing in both ears, otalgia, discharge, vertigo, noise exposure, prior ear surgery, prior ear infections, and prior ototoxic medications.

**Figure 4:**
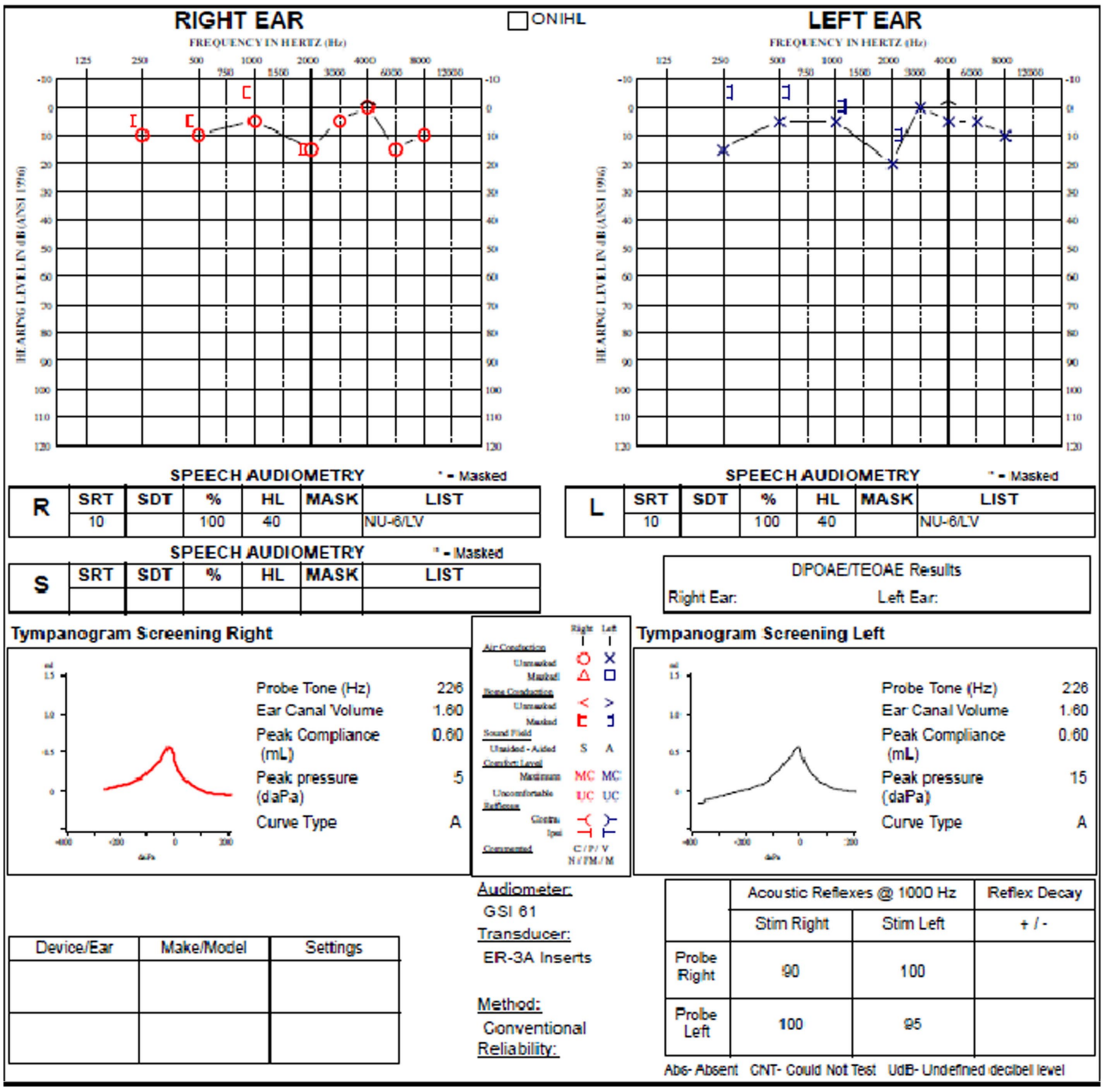
Audiogram of subject with left SSCD demonstrates relatively modest air-bone gap in the left ear.

The review of his audiogram (Figure 4) demonstrated normal hearing on the right with bone conduction hypersensitivity at 1000 Hz; in the left ear, there was mild conductive hearing loss with bone conduction hypersensitivity with negative thresholds at 250 and 500 Hz. Speech reception threshold on the right was 10 dB with a word recognition score of 100%, and speech reception threshold on the left was 10 dB with a word recognition score of 100%. Tympanograms were type A bilaterally, and acoustic reflexes were present in all conditions.

**Figure 5:**
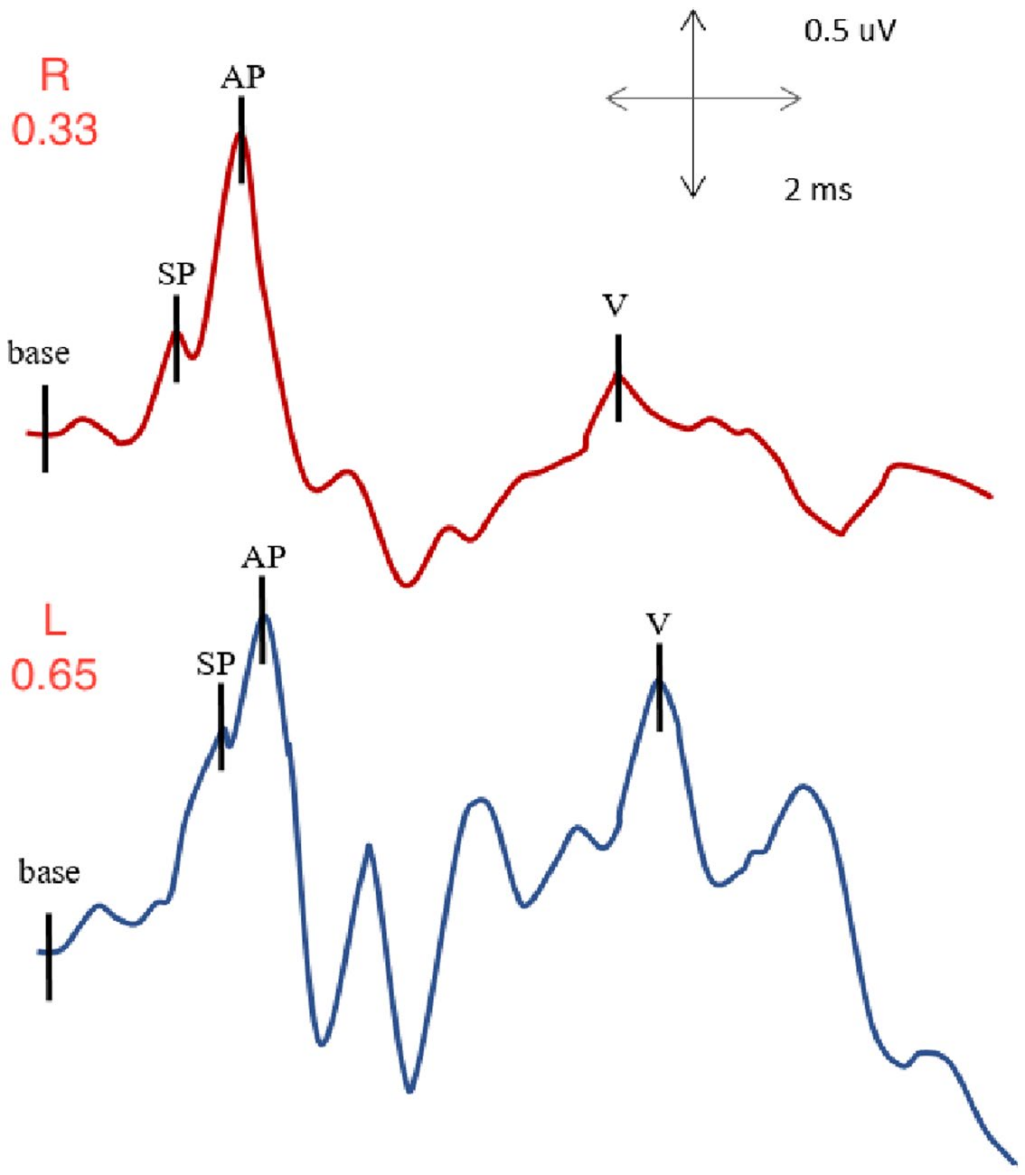
EcochG from right and left ears of subject with left SSCD: normal SP/AP value for the right ear (0.33) and elevated SP/AP value for the left, involved ear (0.65).

Electrocochleography (Figure 5) was carried out using a set of surface electrodes along with a hydrogel tympanic membrane electrode introduced under microscopic visualization of the ear canals and tympanic membranes, as previously described. Clicks were used as stimuli.

With right ear stimulation, the summating potential to action potential ratio was within normal limits with a value of 0.33. The auditory brainstem response I-V interpeak latency, also available in such recording was also evaluated, and was within normal limits with a value of 3.94 ms.

With left ear stimulation, the summating potential to action potential ratio was elevated with an abnormal value of 0.65. We also evaluated the auditory brainstem response I-V interpeak latency, which was within normal limits with a value of 4.02 ms.

**Figure 6:**
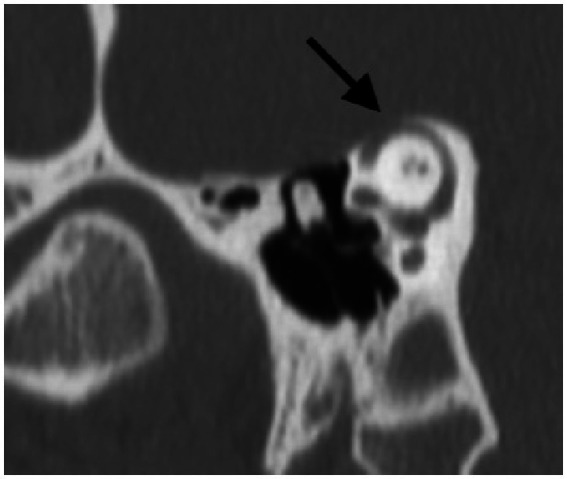
Poschl view of left SSCD shows clear absence of bone over the left superior semicircular canal.

A CT scan was obtained subsequently and demonstrated a bony dehiscence involving the left superior semicircular canal, and relative thinning involving the bony plate of the right superior semicircular canal but no dehiscence (Figure 6).

In summary, this patient presented with predominantly left ear symptoms that raised the suspicion of left superior semicircular canal dehiscence. CT imaging confirmed the presence of dehiscence of the left superior canal. He subsequently underwent surgical repair to plug the left superior semicircular canal. He did well post-operatively, and his disturbing autophony and other symptoms resolved immediately. Follow-up testing within 1 year of surgery demonstrated intact hearing in the left, operated ear, and a normal SP/AP value in the operated ear.

## Discussion and conclusion

The results of this report demonstrated that the EcochG SP/AP ratio was abnormal in the dehiscent ear of all of the patients with bilateral or unilateral SSCD. When the findings were examined for patients with bilateral SSCD in which symptoms localized to one ear, the SP/AP ratio trended toward being higher for that ear. This physiological change in the EcochG SP/AP ratio was observed to normalize in real time during the surgical procedure, intraoperatively, as previously reported by our group (5, 8, 9). This study did not address intraoperative and postoperative changes in SP/AP values in patients with SSCD as the purpose was to determine the diagnostic utility of EcochG and its relationship with air-bone gap in patients with this condition. One of the limitations of the current study is that it is a retrospective study without a corresponding control group for our experimental group. However, it is of note that 11 normal ears from 11 out of 20 subjects with unilateral SSCD were included in the data analysis. Additionally, the use of a multiplanar reformatted CT has been shown in previous studies to have a negative predictive value of 100%. Accordingly, the chance that the ears assigned as “negative” in our study have an undiagnosed SSCD is extremely low.

The EcochG is a response recorded from the tympanic membrane that represents the evoked responses from both the cochlea and the auditory nerve in response to an auditory stimulus. The response occurs in approximately the first 3 ms following the presentation of the stimulus. Although the mode of stimulation and recording parameters can vary the response, it is typically composed of the peripheral end-organ responses that are the cochlear microphonic and summating potential and neural response referred to as the compound action potential of the cochlear nerve. In the current study, the EcochG was recorded using a commercially available tympanic membrane electrode.

The response properties of the SP are that it is a direct current response, can be represented as a positive or negative shift in polarity when measured from the pre-stimulus baseline, and can occur simultaneously with the cochlear microphonic. The response is often recorded using an alternating polarity to cancel out the CM. The origin of the SP is believed to be derived from both the inner and outer hair cells and represents the non-linear mechanical properties of the cochlea. For a brief, transient stimulus, the SP waveform will typically occur immediately before the AP. The AP is commonly displayed as a negative deflection and represents the simultaneous activation of the auditory nerve fibers. This response, although opposite in polarity, is the Wave I observed during an auditory brainstem response recording.

The SP response can vary significantly in amplitude, and an increase in amplitude as measured against the AP amplitude has been suggested as an indication of endolymphatic hydrops commonly associated with the disorder referred to as Meniere’s disease (MD). This increase in amplitude of the SP has been set forth as being due to increased static displacement of the basilar membrane toward the scala tympani in the cochlear. The underlying mechanism for this increased movement has been reported to be due to an increase in the hydrostatic pressure above the basilar membrane in the cochlea. This phenomenon presents clinically when the SP and AP are recorded in patients with active MD. As many as two-thirds of patients with MD have been suggested to generate abnormal SP/AP ratios. This has also been shown in animal models that reported significant hydromechanical alterations in the scala media and subsequently explained the enhanced SP relative to the AP (12). Interestingly, the SP/AP ratio could be returned to normal by removing perilymph using suction through a fistula created surgically in the cochlea (13). A third window condition such as SSCD has been shown to mimic the enhanced SP/AP ratio observed in endolymphatic hydrops by altering the hydrostatic properties of the cochlea. The physiological mechanism for this effect of a third window on the SP/AP ratio may be that the pressure in the scala tympani and scala vestibuli is reduced creating an endolymphatic differential pressure. During surgical exposure and drilling of the dehiscence to access the perilymph compartment, a significant drop in perilymph pressure was observed. The physiological response from this introduction of a third window to the cochlear system was explained as causing a reduction in the perilymphatic pressure and a corresponding increase in the endolymphatic pressure. The effect of this change in pressure would push the basilar membrane toward the scala tympani and would result in an enhanced SP when EcochG techniques were used for recording. This is a common observation during EcochG intraoperative monitoring of the dehiscence repair. Effective plugging of the dehiscence is more often than not associated with the immediate reduction of the SP/AP ratio that brings it into the normal range (5, 8). All ears with CT-confirmed SSCD in this cohort generated elevated SP/AP ratios exceeding our stated normal of 0.4. None of the ears with intact (even thinning) bony covering exhibited increased SP/AP values above 0.4. It is of note, that some of the ears with thin, but intact bone, and negative (normal) SP/AP ratio, were associated with abnormal C-VEMP results, further supporting the superiority of the EcochG as a diagnostic tool for SSCD. According to these findings, we set forth that SSCD should be strongly considered as being in the differential diagnosis when there is an observed enhanced SP/AP ratio and symptoms that would suggest a third window disorder (e.g., Tullio’s phenomenon).

Our results presented in this study continue to illustrate how the EcochG has a high sensitivity for detecting SSCD independent of the necessity of active patient participation, that may be challenging to some, as is the case with the cVEMP. Tympanic EcochG is not associated with the anxiety and discomfort of a transtympanic needle, and it provides excellent response resolution. As a result, a much wider proportion of the patient population is appropriate for this diagnostic test, if necessary. This is not to be confused with the so-called Tip-trode, ear canal EcochG measurement which has a much poorer resolution and tends to be very inconsistent. The EcochG offers a number of advantages over the VEMP for identifying active SSCD. This includes not requiring EMG monitoring, patient fatigue associated with activating the sternocleidomastoid muscle, and a significant absence of responses in patients over 65 years. Our institutional experience has evaluated and diagnosed with EcochG approximately 3,000 patients. Our EcochG procedures have been shown to be well tolerated by older pediatric patients and can be done in the outpatient setting and inpatient setting alike. As such, the EcochG has a great clinical utility in the operating room for patients with SSCD. While VEMP cannot be performed under general anesthesia, the EcochG has no such limitation. In the operating room setting during an SSCD repair, the EcochG can document immediate changes in the SP/AP ratio and confirm that the plugging or resurfacing of the dehiscence is successful.

This report adds new information to what we have learned till now using the EcochG technique to assess and manage patients with SSCD. As previously noted, although there was a positive trend in frequency and audiometric ABGs, our results failed to reach significance. Furthermore, the non-significant *p*-values for the correlations between ABG and SP/AP ratios in ears with confirmed SSCD suggests that the association of ABGs is similar for ears with and without SSCD. It is possible that where the sample is larger, there is a significant effect. The determination of ABG is a purely subjective measure as opposed to the SP/AP ratio derived from appropriately carried out EcochG. The advantages of the EcochG over tests such as audiometry and VEMP are that it is a purely objective measure and not dependent upon the patients’ level of focus and overall participation.

## Data availability statement

The original contributions presented in the study are included in the article/supplementary material, further inquiries can be directed to the corresponding authors.

## Ethics statement

Ethical approval was not required for the study involving humans in accordance with the local legislation and institutional requirements. Written informed consent to participate in this study was not required from the participants or the participants’ legal guardians/next of kin in accordance with the national legislation and the institutional requirements.

## Author contributions

PK: Conceptualization, Investigation, Methodology, Supervision, Writing – original draft, Writing – review & editing. MC: Data curation, Formal analysis, Investigation, Writing – original draft, Writing – review & editing. DM: Data curation, Investigation, Writing – review & editing.
